# Leishmanicidal Evaluation of Tetrahydroprotoberberine and Spirocyclic Erythrina-Alkaloids

**DOI:** 10.3390/molecules19055692

**Published:** 2014-05-05

**Authors:** Daniel R. Callejon, Thalita B. Riul, Luis G. P. Feitosa, Thais Guaratini, Denise B. Silva, Achyut Adhikari, Ram L. (S.) Shrestha, Lucas M. M. Marques, Marcelo D. Baruffi, João L. C. Lopes, Norberto P. Lopes

**Affiliations:** 1 Núcleo de Pesquisa em Produtos Naturais e Sintéticos (NPPNS), Faculdade de Ciências Farmacêuticas de Ribeirão Preto (FCFRP), Universidade de São Paulo (USP), Av. Café s/n, 14040-903, Ribeirão Preto, SP, Brazil; E-Mails: daniel_callejon@yahoo.com.br (D.R.C.); luis@lychnoflora.com.br (L.G.P.F.); thaisguaratini@yahoo.com.br (T.G); denisebrentan@gmail.com (D.B.S.); adhikarimine@yahoo.com (A.A.); lucasmauriz@yahoo.com.br (L.M.M.M.); joaoluis@usp.br (J.L.C.L.); 2 Lychnoflora Pesquisa e Desenvolvimento em Produtos Naturais LTDA, Ribeirão Preto 14030-090, SP, Brazil; 3 Departamento de Análises Clínicas, Faculdade de Ciências Farmacêuticas de Ribeirão Preto (FCFRP), Universidade de São Paulo (USP), Av. Café s/nº, 14040-903, Ribeirão Preto, SP, Brazil; E-Mails: tbriul@hotmail.com (T.B.R.); mdbaruff@fcfrp.usp.br (M.D.B.); 4 H. E. J. Research Institute of Chemistry, ICCBS, University of Karachi, Karachi 75270, Pakistan; 5 Amrit Science Campus, Tribhuvan University, Kathmandu, Nepal; E-Mail: Swagatstha@hotmail.com

**Keywords:** *Leishmania amazonensis*, spirocyclic erythrina-alkaloids, tetrahydro-protoberberine-type alkaloids, *Corydalis govaniana*, *Erythrina verna*

## Abstract

Leishmaniasis is one of the World’s most problematic diseases in developing countries. Traditional medicines to treat leishmaniasis have serious side effects, as well as significant parasite resistance problems. In this work, two alkaloids **1** and **2** were obtained from *Corydalis govaniana* Wall and seven alkaloids **3**–**9**, were obtained from *Erythrina verna*. The structures of the compounds were confirmed by mass spectrometry and 1D- and 2D-NMR spectroscopy. The leishmanicidal activity of compounds **1**–**9** against *Leishmania amazonensis* was tested on promastigote forms and cytotoxicity against J774 (macrophage cell line) was assessed *in vitro*. Compound **1** showed potent activity (*IC*_50_ = 0.18 µg/mL), compared with the standard amphotericin B (*IC*_50_ = 0.20 µg/mL). The spirocyclic erythrina-alkaloids showed lower leishmanicidal activity than dibenzoquinolizine type alkaloids.

## 1. Introduction

Neglected tropical diseases (NTD) are caused by several infectious agents and cause high levels of mortality and morbidity worldwide [1]. Around one billion people are affects by NTD or are exposed to the causative agents of these diseases [2]. Unfortunately, funding and research focus for the development of therapeutics and prevention strategies applicable to these neglected diseases are insufficient [2,3]. The modification of this scenario represents a crucial challenge to advance the human health.

Leishmaniasis is one type of NTD that is considered as a major health problem worldwide. This disease is caused by protozoa belonging to the *Leishmania* genus, and can present a broad spectrum of symptoms such as cutaneous, lesion and fatal visceral infections [4]. The World Health Organization (WHO) estimated that diseases caused by *Leishmania* sp. threaten 350 million people and are responsible for about 2 million clinical cases each year in 88 countries [5]. The countries with higher prevalence of leishmaniasis are subtropical and developing countries, for example, India, Sudan, Bangadesh, Nepal and Brazil [5].

Currently, most available drugs against leishmaniasis have high toxicity, require long treatment regimens and are costly. These problems reduce treatment adherence by patients and increase the emergence of resistant strains [6]. In addition, there are no efficient vaccine candidates against this parasite [7]. The current treatment of leishmaniasis is based on pentavalent antimony compounds, which include sodium stibogluconate (Pentostam^®^) and meglumine antimoniate (Glucantime^®^). These compounds are widely prescribed despite their severe side effects in the heart, kidney, pancreas and liver, high cost, difficult administration and development of parasite resistance [8]. Other drugs such as amphotericin B, pentamidine and metilfosineare are used in leishmaniasis treatment, but their clinical applications are limited because of their toxicity, adverse side effects and high cost [8].

Nature has provided an innumerable number of biological activity compounds and plants are still the predominant source of drug leads [9]. Two recently reported reviews survey the importance and the potential of plant natural products as antiprotozoal leads for NTDs [9,10]. In total more than 850 compounds were listed, and the activity against at least one NTD verified [9,10]. In this compilation, alkaloids represent around of 20% of the total of compounds with antiparasite effect, and the major representative compounds were quinolines, isoquinolines, indoles, steroids and diterpenes, but other classes are also represented by few examples [9,10]. In a recent review, many alkaloids exhibiting leishmanicidal activity were reported [11]. A detailed analysis of the published active structures in addition of a specific literature review revealed no occurrence of erythrina-alkaloids anti-protozan analysis [12]. These alkaloids are common in *Erythrina* species, which belong to the Fabaceae family occurring tropical and subtropical forests [13,14]. Until now more than one hundred spirocyclic erythrina-alkaloids were reported and the major pharmacological activities were related to as anxiolytic, sedative and central nervous system depression [14,15,16,17,18,19,20,21,22,23]. Tetrahydroprotoberberine-type alkaloids, typical compounds of *Corydalis* species, also show important central nervous system actions in addition to anti-malarial effects [24,25], but data about leishimanicidal or trypanocidal activity were not reported. Species of *Corydalis* (family Papaveraceae) have occurrence predominantly in Asia and in mountainous regions of Eastern Africa [26]. Considering the importance of alkaloids for the search of the new biological structures activities against NTD and the reduced (or even absent) amount of data about leishmanicidal activities of spirocyclic erythrina and tetrahydroprotoberberine-type alkaloids, the aim of this work was to contribute with information about the leishmanicidal activity from these alkaloids. So, the phytochemical investigation from two commercial cultivars of *E. verna* was performed, and seven spirocyclic erythrina and two previous isolated tetrahydroprotoberberine-type alkaloids [27] were evaluated against *Leishmania amazonensis*.

## 2. Results and Discussion

### 2.1. Isolation and Structural Determination of Alkaloids

**Figure 1:**
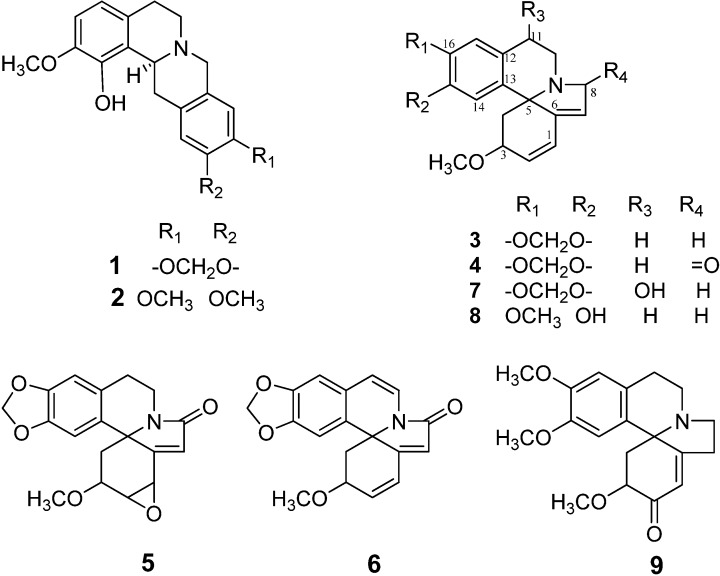
Structures of alkaloids **1**–**9**.

*Corydalis govaniana* extract was fractionated based on our previous experience. At this time the phytochemical procedures allowed the isolation of alkaloids **1** and **2** [27]. The compounds erythraline (**3**) and 8-oxoerythraline (**4**) were previously isolated from *Erythrina verna* by us and reported (Figure 1) [28].

The ^1^H- and ^13^C-NMR spectra of compounds **4**–**6** showed some similar signals, such as signals that confirmed the presence of methylenedioxy, methoxyl and carbonyl groups. In the mass spectrum of compound **5**, an ion at *m/z* 328.1190 [M+H]^+^ was observed, confirming the molecular formula C_18_H_18_NO_5_ (error: 3.3 ppm, calcd. for C_18_H_18_NO_5_^+^ 328.1179). The signals observed in ^1^H-NMR spectrum at δ 3.58 (H-2) and 4.07 (H-1) with *J* = 4.0 Hz, together with their carbons δ 57.7 (C-2) and 51.0 (C-1), suggested the presence of an epoxide ring. From all the spectroscopic data, the structure of compound **5** was found to be 8-oxoerythraline epoxide. All the physical and spectroscopic data unambiguously matched with reported compound from *Erythrina x bidiwillii* [29] in the only study that reported its isolation.

For compound **8**, an ion at *m/z* 300.1594 [M+H]^+^ was observed in the HRESI spectrum, and its molecular formula was determined to be C_18_H_21_NO_3_ (error: 2.0 ppm, calcd. for C_18_H_22_NO_3_^+^ 300.1600). The ^1^H-NMR spectrum revealed five olefinic hydrogen signals, including two aromatic hydrogens at δ_H_ 6.86 and 6.64 and three signals related to a conjugated diene [δ_H_ 6.54 (H-1), 6.01 (H-2) and 5.71 (H-7)]. Furthermore, the signals at δ_H_ 3.31 and 3.89 indicated the presence of two methoxy groups. The presence of a methoxyl at C-16 position was confirmed by the correlations obtained in 2-D NMR experiments HMBC and NOESY (Supplementary Material). The signal at δ_H_ 6.64 (H-17) showed correlation with the C-11 in the HMBC spectrum, while the signal at δ_H_ 6.86 (H-14) showed correlation with C-5. The NOESY spectrum revealed the correlation between signal at δ_H_ 6.86 (H-14) and 4.06 (H-3), which was already reported for erythrina-alkaloids from NOE *diff* experiments [30]. Moreover, the correlation between δ_H_ 3.89 (OCH_3_) and H-17 was also observed in the NOESY spectrum. Therefore, the compound **8** was determined to be erysovine and all data was in agreement with published reports, except the inverted assignment between aromatic hydrogens H-14 and H-17 and between C-15 and C-16 [31,32]. The ^1^H- and ^13^C-NMR data of erysovine published in the literature were assigned through the comparisons between erythrina derivatives [31,32]. However, a safer structural determination and attribution of chemical shifts was only obtained from the homonuclear and heteronuclear correlations observed in 2D-NMR spectra of alkaloid **8**. All spectroscopic data of spirocyclic erythrina-alkaloids isolated agree with the previously published data [28,29,31,32,33,34,35].

### 2.2. Evaluation of Leishmanicidal Activity

The leishmanicidal activity of the different alkaloids was evaluated and is shown in Table 1. The results demonstrated that **1**, a tetrahydroprotoberberine-type alkaloid obtained from *Corydalis govaniana*, has a significant leishmanicidal activity on promastigote forms of *L. amazonensis*. In addition, the cytotoxicity against macrophage cell lines of **1** was low and the selectivity index (SI) calculated (CC_50_ drug/IC_50_ drug) was 284.55, suggesting that it has no toxicity effect to mammalian cells. The compound **2**, other tetrahydroprotoberberine-type alkaloid, didn’t show significant toxicity against *L. amazonensis* (*IC*_50_ > 1,000). Comparing the chemical structures of alkaloids **1** and **2**, it is possible to observe the modification in relation to methylenedioxy moiety, which is not present in **2**. So, this observation may suggest that the methylene dioxide is important for the leishmanicidal activity of the alkaloid **1**, but further investigations must be performed to confirm this hypothesis.

All spirocyclic erythrina-alkaloids showed lower leishmanicidal activity than alkaloid **1** (Table 1 and Supplementary Material). They also showed very low SI, suggesting a possible cytotoxicity to mammalian cells at the concentration toxic to promastigote forms of *L. amazonensis*. The most active erythrina-alkaloid was **7**, showing an *IC*_50_ of 39.53 µg/mL. The absence of a hydroxyl in position 11 (alkaloid **3**) resulted in a decrease of the leishmanicidal activity (*IC*_50_ 65.27 µg/mL), at the same way the absence of methylenedioxy moiety from position 3 (alkaloid **8**) also resulted in a decrease of activity (*IC*_50_ > 1,000). Besides, the presence of a carbonyl at carbon 8 (alkaloid **4**) decreased the activity significantly (*IC*_50_ > 1,000), as observed from the comparison with the alkaloid **3**. For the alkaloids **5 **and **6**, derivatives from 8-oxoerytraline (**4**), it was possible to verify that addition of an epoxide (at C-1 and C-2 positions) and a double bond at C-10 and C-11 improved the activity when compared with results obtained for **4**.

**Table 1:** Leishmanicidal activity of compounds **1**–**9**, against *Leishmania amazonensis*.

Compound	*L. amazonensis*	Macrophage cells (J774)	SI
	*IC*_50_ (µg/mL)	*CC*_50_ (µg/mL)	
1	0.18	51.22	284.55
2	>1000	96.56	ND
3	65.27	69.33	1.06
4	>1000	>1000	ND
5	48.17	47.66	0.98
6	71.53	58.97	0.82
7	39.53	59.97	1.52
8	>1000	>1000	ND
9	>1000	>1000	ND
Amphotericin B	0.20	NT	NT

SI: Selectivity Index = CC_50_ drug/IC_50_ drug; ND: Not determined; NT: not tested.

Our studies suggested that some chemical features in the alkaloids studied are relevant for the leishmanicidal activity. For the tetrahydroprotoberberine-type and erytrinia-alkaloids, a methylenedioxy moiety was fundamental to activity. Some isoquinoline alkaloids with methylenedioxy moieties, such as *O*-methylmoschatoline and liriodenine, induced significant cell death of *L. brasiliensis*, and the higher activity was correlated with the presence of this moiety [11] as well as described in other studies [36]. Montenegro *et al*. verified that alkaloids with methylenedioxy moieties decreased parasite burden like amphotericin B. However, the replacement of a methoxy group decreased the leishmanicidal activity [37]. The leishmanicidal activity observed for the erythrina-alkaloids was not high, but important structural chemical features that influence their activities were recognized, such as the methylenedioxy and a hydroxyl substituent at C-11.

## 3. Experimental Section

### 3.1. General

Column chromatography was carried out on silica gel (300–400 mesh, Qingdao Marine Chemical Ltd., Qingdao, China). Thin layer chromatography (TLC) was performed on TLC silica gel 60 F254 plates. Preparative HPLC-DAD was performed on a Shimadzu LC-6AD system equipped with a Shimadzu UV/VIS SPD-M20A DAD detector. NMR spectra were recorded on Bruker 400 and 500 MHz instruments. The chemical shifts were recorded in ppm relative to tetramethylsilane and with the solvent (CDCl_3_) resonance as the internal standard. MS spectra were obtained on a Waters Acquity^TM^ ESI-TQ and Bruker microTOFq II– ESI-TOF spectrometers. The samples were solubilized in methanol and injected into the spectrometers.

### 3.2. Isolation of Alkaloids

#### 3.2.1. Plant Materials

The whole plant of *Corydalis govaniana* Wall. was collected from Langtang, Rashuwa, Nepal, and identified by Mr. Sanjiv Kumar Rai, Taxonomist, Department of Plant Resources, Thapathali, Kathmandu, Nepal. A voucher specimen, CG-207, has been deposited in Central Department of Botany, Tribhuvan University, Kirtipur, Kathmandu, Nepal. The powdered bark of *E. verna* was acquired from two Brazilian commercial producers, identified as A (6.0 Kg) and B (4.0 Kg).

#### 3.2.2. Extraction and Isolation

The extraction and isolation of alkaloids from *Corydalis govaniana* was previously published [27]. The procedures of extraction and isolation of alkaloids erythraline (**3**) and 8-oxoerythraline (**4**) from *E. verna* (commercial producer A) was previously published [28]. Erythraline was submitted to Mn(salen) oxidation procedure and the alkaloid 8-oxoerythraline (**4**) was isolated from Mn(salen) oxidation reactions by preparative HPLC-DAD [28].

The bark of *E. verna* (commercial producer B) was submitted to extraction by percolation with ethanol. The ethanolic extract was subjected to acid-base extraction to obtained alkaloid fraction. The alkaloid fraction of A was subjected to column chromatography (CC) in silica gel using hexane/ethyl acetate (8:2, 7:3, 6:4, 4:6, 1:9) to yield 155 fractions. The fractions 9–25 were purified by preparative HPLC-DAD to obtain **5** (17.8 mg) and **6** (13.7 mg). Compound **5** was isolated as a mixture with **4**. The fractions 136–144 obtained from CC of F2 A were subjected to preparative TLC in silica gel GF_254_ with ethyl acetate-methanol (7:3) as eluent. From this procedure, compound **7** (12.3 mg) and compound **8** (10.1 mg) were obtained.

The barks of *E. verna* obtained from commercial producer B was also extracted according to the previously described method [38]. Thereby, the barks of B (3.4 Kg) were extracted by percolation with ethanol, yielding 8.4% (285.6 g) of ethanolic extract. The ethanolic extract (266.0 g) subjected to acid-base extraction to yield 0.17% (0.45 g) of alkaloid fraction. Then, a mass of 0.40 g of alkaloid fraction B was subjected to preparative HPLC-DAD in a C-18 column (Shim-pack Prep-ODS, 5 µm, 20 mm × 25 cm, Shimadzu), flow rate 9 mL/min and acetonitrile (B) and water (A) both with TFA 0.02% (*v/v*) as solvents. The elution profile was 0–10 min — 10% B, 10–35 min — 10%–30% B, 35–55 min — 30%–60% B and 55–60 min — 60%–100% B. This purification yielded compound **9** (52.0 mg).

All compounds isolated from alkaloid fractions of A and B were analyzed by HRESI-MS (microTOFq II– ESI-TOF) in positive ionization mode. The samples are submitted to direct infusion in mass spectrometer, using N_2_ as nebulizer gas, drying gas at 200 °C and pressure of 0.4 Bar, capillary voltage of 4,500 V.

#### 3.2.3. Spectral Data of Alkaloids

Data for *Erythraline* (**3**) and *8-oxoerythraline* (**4**) was previously published [28]. *8-Oxoerythraline epoxide* (**5**): ^1^H-NMR (CDCl_3_, 400 MHz) δ_H_ 7.19 (s, H-7), 4.07 (d, *J* = 4.0 Hz, H-1), 6.60 (s, H-14), 6.44 (s, H-17), 5.87 (d, *J* = 1.3, -OCH_2_O-), 5.85 (d, *J* = 1.3, -OCH_2_O-), 3.58 (dd, *J* = 4.0 Hz, H-2), 3.53 (ddd, *J* = 3.5, 7.1 and 12.4 Hz, H-10), 3.43 (dd, *J* = 4.9 and 10.5 Hz, H-3), 3.66 (ddd, *J* = 6.6, 10.6 and 12.4 Hz, H-10), 3.34 (s, 3-OCH_3_), 3.04 (ddd, *J* = 7.1, 10.6 and 15.8 Hz, H-11), 2.87 (ddd, *J* = 3.5, 6.6 and 15.8 Hz, H-11), 2.54 (dd, *J* = 4.9 and 12.6 Hz, H-4), 1.62 (dd, *J* = 10.5 and 12.6 Hz, H-4).^13^C-NMR (CDCl_3_, 100 MHz) δ_C_ 169.4 (C-8), 158.4 (C-6), 146.9 (C-15), 145.9 (C-16), 130.3 (C-13), 128.3 (C-7), 128.2 (C-12), 109.5 (C-17), 109.0 (C-14), 101.1 (-OCH_2_O-), 73.8 (C-3), 68.0 (C-5), 58.3 (3-OCH_3_), 57.7 (C-2), 51.0 (C-1), 42.2 (C-10), 38.1 (C-4), 27.8 (C-11). HRESI-MS (positive mode) *m/z* 328.1190 [M+H]^+^ (error: 3.3 ppm, calcd. for C_18_H_18_NO_5_^+^ 328.1179).

*Crystamidine* (**6**): ^1^H-NMR (CDCl_3_, 400 MHz) δ_H_ 6.96 (dd, *J* = 2.5 and 10.2 Hz, H-2), 6.90 (d, *J* = 7.3 Hz, H-10), 6.74 (s, H-17), 6.70 (s, H-14), 6.36 (dl, *J* *=* 10.2 Hz, H-1), 6.15 (d, *J* = 7.3 Hz, H-11), 6.13 (s, H-7), 5.99 (d, *J* = 1.4, -OCH_2_O-), 5.95 (d, *J* = 1.4, -OCH_2_O-), 3.71 (m, H-3), 3.31 (s, 3-OCH_3_), 2.73 (ddl, *J* = 5.1 and 11.3 Hz, H-4), 1.43 (dd, *J* = 10.4 and 11.3 Hz, H-4). ^13^C-NMR (CDCl_3_, 100 MHz) δ_C _169.2 (C-8), 155.9 (C-6), 147.3 (C-16), 146.6 (C-15), 138.2 (C-2), 126.3 (C-12), 125.3 (C-13), 123.6 (C-1), 120.3 (C-10), 119.8 (C-7), 113.9 (C-11), 107.0 (C-17), 104.0 (C-14), 101.4 (-OCH_2_O-), 74.5 (C-3), 66.2 (C-5), 56.4 (3-OCH_3_), 42.7 (C-4). HRESI-MS (positive mode) *m/z* 332.0897 [M+H]^+^ (error: 0.6 ppm, calcd. for C_18_H_15_NO_4_Na^+^ 332.0899).

*Erythrinine* (**7**): ^1^H-NMR (CDCl_3_, 400 MHz) δ_H_ 6.99 (s, H-17), 6.81 (s, H-14), 6.56 (dd, *J* = 10.2 and 2.2 Hz, H-1), 6.0 (dd, *J* = 10.2 Hz, H-2), 5.96 (d, *J* = 1.4, -OCH_2_O-), 5.93 (d, *J* = 1.4, -OCH_2_O-), 5.75 (m, H-7), 4.74 (dd, *J* = 4.5 Hz, H-11), 3.97 (m, H-3), 3.87 (m, H-8), 3.59 (dd, *J* = 4.7 Hz, H-8), 3.34 (s, 3-OCH_3_), 3.00 (dd, *J* = 4.2 Hz, H-10), 2.39 (dddd, *J* = 5.4 and 11.5 Hz, H-4), 1.80 (m, H-4). ^13^C-NMR (CDCl_3_, 100 MHz) δ_C_ 147.2 (C-15), 146.7 (C-16), 142.1 (C-6), 131.5 (C-13), 131.2 (C-2), 130.2 (C-12), 125.3 (C-1), 123.5 (C-7), 107.3 (C-17), 105.8 (C-14), 101.0 (-OCH_2_O-), 76.0 (C-3), 66.8 (C-5), 64.9 (C-11), 59.2 (C-8), 51.6 (C-10), 56.1 (3-OCH_3_), 41.0 (C-4). HRESI-MS (positive mode) *m/z* 314.1403 [M+H]^+^ (error: 3.5 ppm, calcd. for C_18_H_20_NO_4_^+^ 314.1392).

*Erysovine* (**8**): ^1^H-NMR (CDCl_3_, 400 MHz) δ_H_ 6.86 (s, H-14), 6.64 (s, H-17), 6.54 (dd, *J* = 10.1 and 2.2 Hz, H-1), 6.01 (dd, *J* = 10.1 Hz, H-2), 5.71 (m, H-7), 4.06 (m, H-3), 3.89 (s, 16-OCH_3_), 3.74 (dd, *J* = 3.0 and 14.4 Hz, H-8), 3.54 (m, H-8), 3.50 (dd, H-10), 3.00 (m, H-10), 3.35 (s, 3-OCH_3_), 2.97 (m, H-11), 2.67 (m, H-11), 2.53 (m, H-4), 1.87 (dd, *J* = 11.0 Hz, H-4). ^13^C-NMR (CDCl_3_, 100 MHz) δ_C_ 145.3 (C-16), 143.6 (C-15), 142.2 (C-6), 131.8 (C-2), 131.6 (C-13), 125.7 (C-12), 125.1 (C-1), 122.3 (C-7), 112.0 (C-14), 110.8 (C-17), 76.0 (C-3), 66.8 (C-5), 56.5 (C-8), 43.5 (C-10), 41.0 (C-4), 24.1 (C-11), 56.1 (3-OCH_3_), 55.8 (16-OCH_3_). HRESI-MS (positive mode) *m/z* 300.1594 [M+H]^+^ (error: 2.0 ppm, calcd. for C_18_H_22_NO_3_^+^ 300.1600).

*Erythratidinone* (**9**): ^1^H-NMR (CDCl_3_, 400 MHz) δ_H_ 6.72 (s, H-17), 6.45 (s, H-14), 6.29 (m, H-1), 3.92 (dd, *J* = 5.5 and 12.6 Hz, H-3), 3.88 (m, H-8), 3.84 (s, 15-OCH_3_), 3.77 (dd, *J* = 8.6 and 4.6 Hz, H-10), 3.72 (s, 16-OCH_3_), 3.58 (m, H-10), 3.24 (s, 3-OCH_3_), 3.11 (m, H-11), 3.07 (m, H-8), 3.05 (m, H-7), 2.75 (m, H-4), 2.69 (*m*, H-7), 2.58 (dd, *J* = 5.5 and 11.6 Hz, H-4). ^13^C-NMR (CDCl_3_, 100 MHz) δ_C_ 158.7 (C-6), 150.4 (C-15), 148.5 (C-16), 126.7 (C-1), 122.6 (C-13), 119.5 (C-12), 112.8 (C-17), 108.6 (C-14), 75.7 (C-3), 68.2 (C-5), 58.6 (3-OCH_3_), 56.3 (16-OCH_3_), 56.1 (15-OCH_3_), 47.0 (C-8), 40.6 (C-10), 38.4 (C-4), 26.9 (C-7), 21.6 (C-11). HRESI-MS (positive mode) *m/z* 330.1690 [M+H]^+^ (error: 4.5 ppm, calcd. for C_18_H_23_NO_4_^+^ 330.1705).

### 3.3. Leishmanicidal Assay

Promastigote forms of *Leishmania amazonensis* were obtained from infected mice and maintained in Schneider’s medium (Sigma-Aldrich, St. Louis, MO, USA) supplemented with 20% fetal bovine serum, 1% l-glutamine, 10 UI penicillin and 10 µg/mL streptomicin at 24 °C. The colorimetric MTT assay (3-(4,5-dimethylthiazol-2-yl)-2,5-diphenyl-tetrazolium bromide) was used to assess the leishmanicidal activity of compounds as previously described by Mossman [39]. Briefly, log phase promastigotes of *Leishmania amazonensis* were added to 96-well tissue culture plates (1.0 × 10^6^ /well) and treated with 40, 20 and 10 µg/mL of each compound previously diluted in Schneider´s medium with dimethyl sulfoxide (DMSO) for 48 h. Each concentration was tested in triplicate. Two independent experiments were performed, and DMSO concentration in wells was not higher than 0.1%. Controls with DMSO and amphotericin B (cell death control — 2 µg/mL) were also performed in each experiment. After treatment, 10 µL of 5 mg/mL MTT solution was added to each well and the plates were incubated by 4 h at 24 °C. The plate was then centrifuged at 700 *×g* for 10 min. The supernatants were discarded and the formazan crystals from viable cells were solubilized with 200 µL of DMSO. The absorbance of each well was read using a spectrophotometer (Spectramax Plus, Molecular Devices, Sunnyvale, CA, USA) at 570 nm.

### 3.4. Macrophages in Vitro Cytotoxic Assay

To evaluate *in vitro* cytotoxic effect of the compounds on mammal cells, macrophages from J774A.1 lineage were used. 10^5^ cells/well were cultured with RPMI-1640 medium with 10% of fetal bovine serum, 1% l-glutamine, 10 UI penicillin and 10 µg/mL streptomycin in 96 well plates in 5% CO_2_ incubator at 37 °C. The macrophages were rinsed with PBS after 24 h in order to remove non-adherent cells, and then treated with 40, 20 and 10 µg/mL of each compound, previously diluted in RPMI-1640 medium with dimethyl sulfoxide (DMSO). The plates were maintained in the 5% CO_2_ incubator at 37 °C, for 48 h. The DMSO concentration in wells was not higher than 0.1%. Macrophages cells viability was determined by the MTT assay, as described above.

## 4. Conclusions

In conclusion, the present study reports for the first time the investigation of the biological activity of erythrina-alkaloids against a Neglected Disease. As previously discussed, there are several examples of alkaloids with higher activity [10] than that observed in this investigation. Our data suggest that non-planar structures containing an uncommon spirocyclic moiety may be responsible for the lower effects. On the other hand, an alkaloid with a benzoisoquinolizidine structure (compound **1**) has significant activity against *L. amazonensis*. In the present study, the activity of these alkaloids was assessed against promastigote forms. Further studies against amastigote forms will have to show whether the compounds under study, especially compound **1**, are also active against this clinically more relevant life stage of the parasite. Compound **1** can be the lead compound for new drug discovery against leishmaniasis, and further *in vivo* studies will be worthy. It is important to consider the potential use of alkaloids in topical formulations to avoid the side effects of existing drugs, such as effects on the central nervous system.

## Supplementary Materials

Supplementary materials can be accessed at: http://www.mdpi.com/1420-3049/19/5/5692/s1.
