# Supplementary material for: Leishmanicidal Evaluation of Tetrahydroprotoberberine and Spirocyclic Erythrina-Alkaloids
Source: Molecules. 2014 May 5;19(5):5692–703. doi: 10.3390/molecules19055692 (PMC6271856; doi:10.3390/molecules19055692)

Supplementary Materials

1. Structural Characterization of Alkaloids

For compound **7**, it was observed the ion *m/z* 314.1403 $[M+H]^+$ in the HRESI spectrum (positive ionization), with is related to the molecular formula $C_{18}H_{19}NO_4$, corresponding to erythrinine. In the ^{1}H NMR spectrum of compound **7** it was observed five signals of olefinic hydrogens with belongs to the conjugated diene hydrogens [δ_H 6.56 (H-1), 6.00 (H-2) and 5.75 (H-7)] and the hydrogens of aromatic ring [δ_H 6.81 (H-14) and 6.99 (H-17)]. The signal at δ_H 3.34 corresponding to a methoxy group. Furthermore, the signals at δ_H 5.93 and 5.95 (*d*, *J* = 1.4 Hz) indicated the presence of methilenodioxy group. The presence of hydroxyl group at C-11 was confirmed mainly due to shift of C-11 compared with the observed to erythraline, suggesting the presence of oxygen atom bond to C-11 of compound **7**. Morevoer, it was observed six methylene carbons in the DEPT 135° spectra of compound **7**, while the DEPT 135° spectra of erythraline showed five methyne carbons. It was also observed four methylene carbons and a methoxy group in the DEPT 135° spectra of compound **7**. The compound **7** was determined erythrinine and all data were agreed with the published [8,24].

The HRESI spectrum in positive ionization of compound **9** showed the ion of *m/z* 330, 1690 $[M+H]^+$ and its molecular formula was determined $C_{18}H_{23}NO_4$, corresponding to erythratidinone. The ^{1}H-NMR spectrum showed three signals of olefinic hydrogens, including two signals of aromatic hydrogens at δ_H 6.45 and 6.72 and the signal of vinylic hydrogen H-1 at δ_H 6.29. The signals at δ_H 3.42, 3.84 and 3.72 indicated the presence of three methoxy groups. The assignments of hydrogens and carbons were obtained by correlations in HMQC spectra. In HMBC spectra was verified correlation between signals at δ_H 3.42, 3.84 and 3.72 with C-3, C-15 and C-16, respectively. The ^{13}C-NMR spectrum showed a signal at δ_C 196.0, suggesting the carbonyl group. The correlation between H-3 and H-4 with the signal at δ_C 196.0 in HMBC spectra indicated the presence of carbonyl in C-2. Twelve resonances carbons was observed in 135° DEPT spectra of compound **9**, including four methynes, five methylenes and three methoxy. Thus, the compound **9** was determined erythratidinone and the ^{1}H-NMR data were agreed with the published.

Figure S1. Correlations observed in HMBC (8 Hz) spectra of erysovine (**8**) [correlations in blue is observed in the NOESY spectrum] and erythratidinone (**9**).

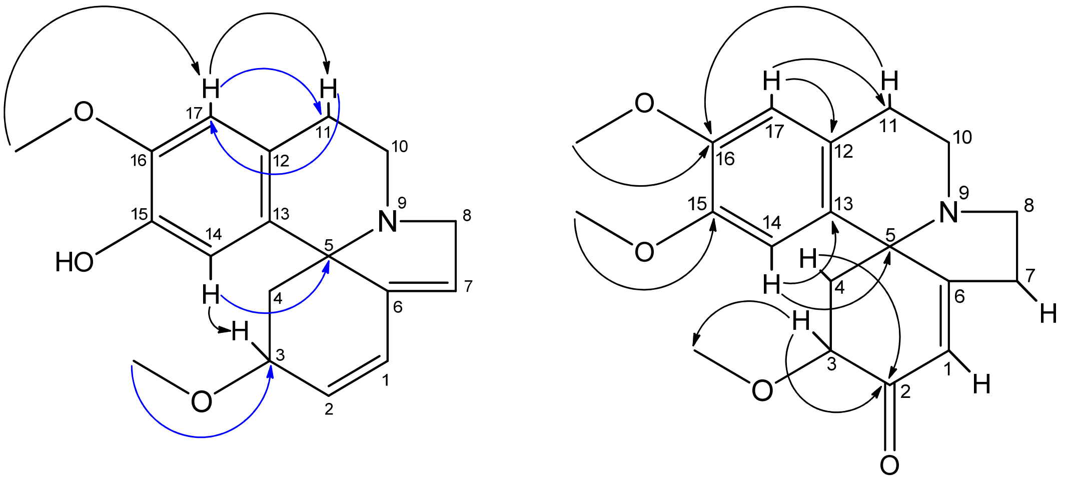

Erysovine (**8**) Erythratidinone (**9**)

2. Effects of Alkaloids in the Cytotoxicity *of L. amazonensis and* J774 Macrophage Cell Line

Figure S2. Effect of compound **1** on cell viability of *L. amazonensis* and J774 macrophage cell line. ANF: Amphotericin B; CPT: Camptothecin; * $p < 0.05$ one-way ANOVA) followed by Bonferroni's test.

Compound 1

L. amazonensis

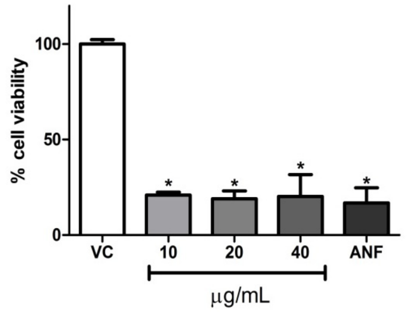

J774 macrophage cell line.

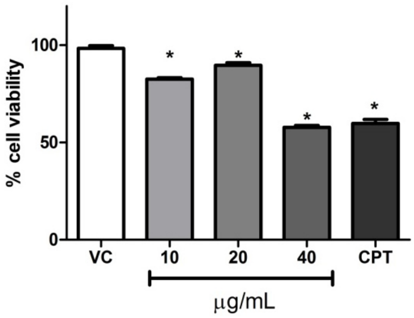

Figure S3. Effect of compound **2** on cell viability of *L. amazonensis* and J774 macrophage cell line. ANF: Amphotericin B; CPT: Camptothecin; * $p < 0.05$ one-way ANOVA) followed by Bonferroni's test.

Compound 2

L. amazonensis

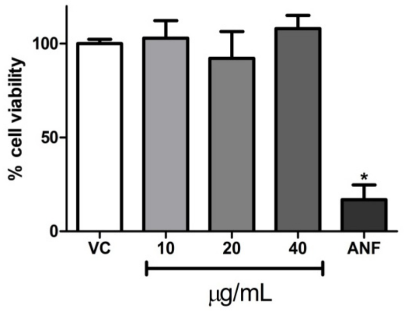

J774 macrophage cell line.

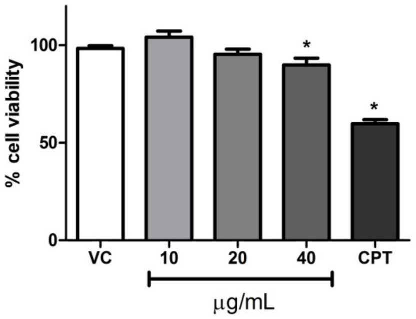

Figure S4. Effect of compound **3** on cell viability of L. amazonensis and J774 macrophage cell line. ANF: Amphotericin B; CPT: Camptothecin; * $p < 0.05$ one-way ANOVA) followed by Bonferroni's test.

Compound 3

L. amazonensis

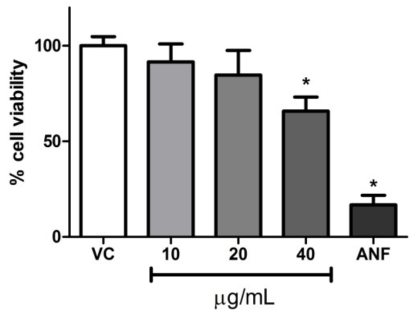

J774 macrophage cell line.

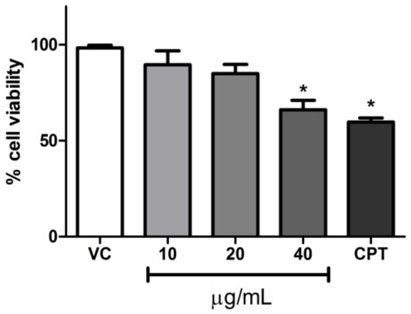

Figure S5. Effect of compound **4** on cell viability of L. amazonensis and J774 macrophage cell line. ANF: Amphotericin B; CPT: Camptothecin; * $p < 0.05$ one-way ANOVA) followed by Bonferroni's test.

Compound 4

L. amazonensis *J774 macrophage cell line.*

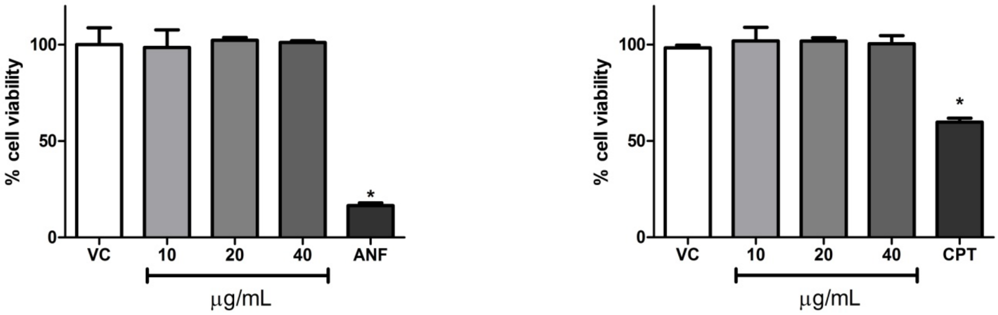

Figure S6. Effect of compound **5** on cell viability of L. amazonensis and J774 macrophage cell line. ANF: Amphotericin B; CPT: Camptothecin; * $p < 0.05$ one-way ANOVA) followed by Bonferroni's test.

Compound 5

L. amazonensis *J774 macrophage cell line.*

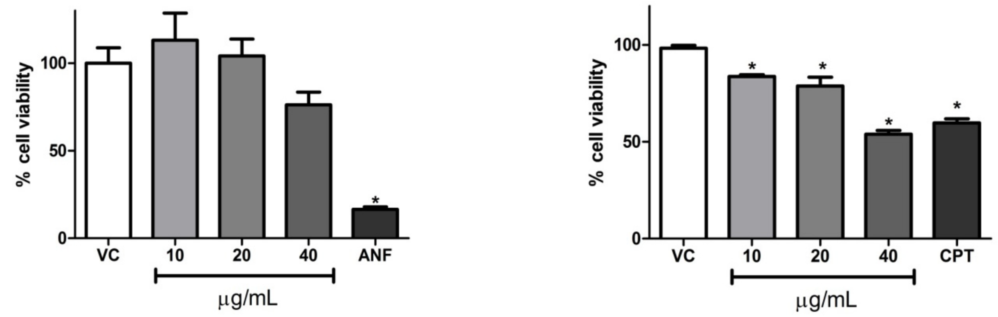

Figure S7. Effect of compound 6 on cell viability of L. amazonensis and J774 macrophage cell line. ANF: Amphotericin B; CPT: Camptothecin; * $p < 0.05$ one-way ANOVA) followed by Bonferroni's test.

Compound 6

L. amazonensis *J774 macrophage cell line.*

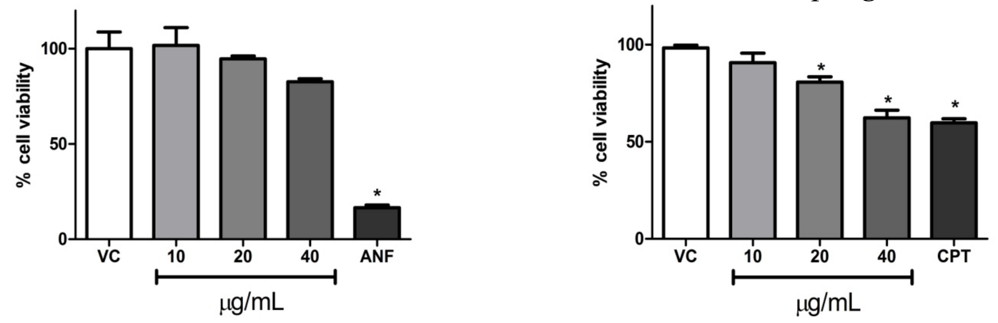

Figure S8. Effect of compound **7** on cell viability of L. amazonensis and J774 macrophage cell line. ANF: Amphotericin B; CPT: Camptothecin; * $p < 0.05$ one-way ANOVA) followed by Bonferroni's test.

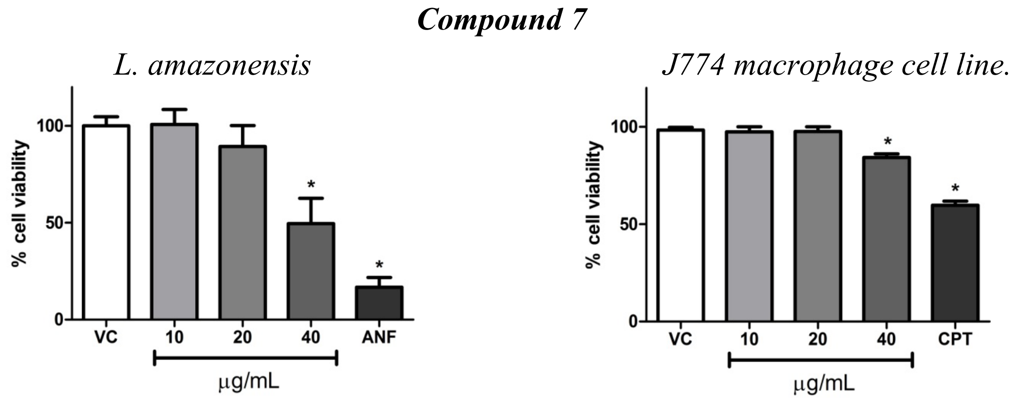

Figure S9. Effect of compound **8** on cell viability of L. amazonensis and J774 macrophage cell line. ANF: Amphotericin B; CPT: Camptothecin; * $p < 0.05$ one-way ANOVA) followed by Bonferroni's test.

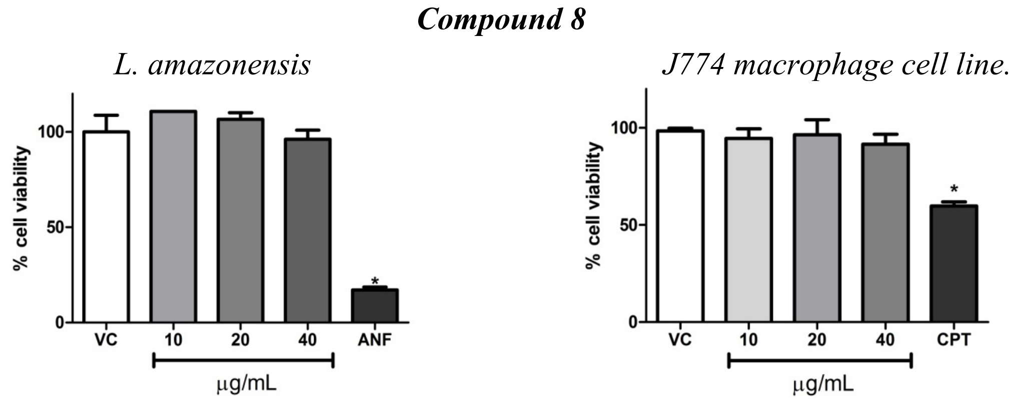

Figure S10. Effect of compound **9** on cell viability of L. amazonensis and J774 macrophage cell line. ANF: Amphotericin B; CPT: Camptothecin; * $p < 0.05$ one-way ANOVA) followed by Bonferroni's test.

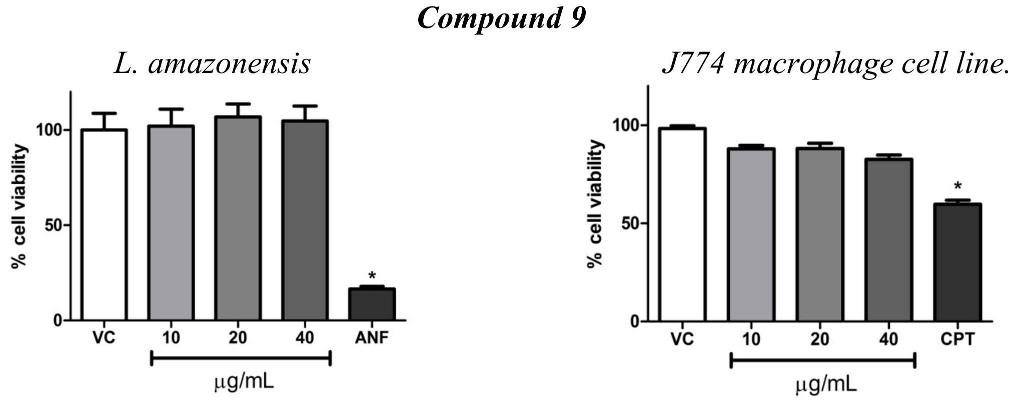

NMR and MS Spectra

Figure S11. ^{1}H-NMR spectrum of erythrinine (**7**) [$CDCl_3$, 400 MHz].

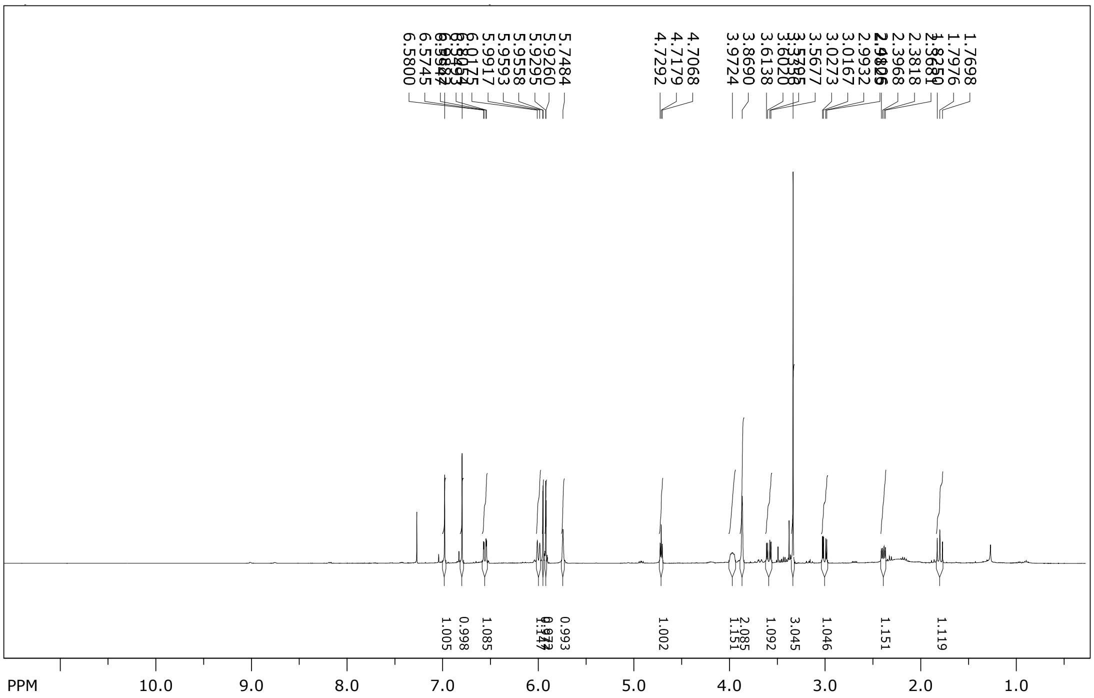

Figure S12. ^{1}H-NMR spectrum of erythrinine (**7**) [$CDCl_3$, 400 MHz].

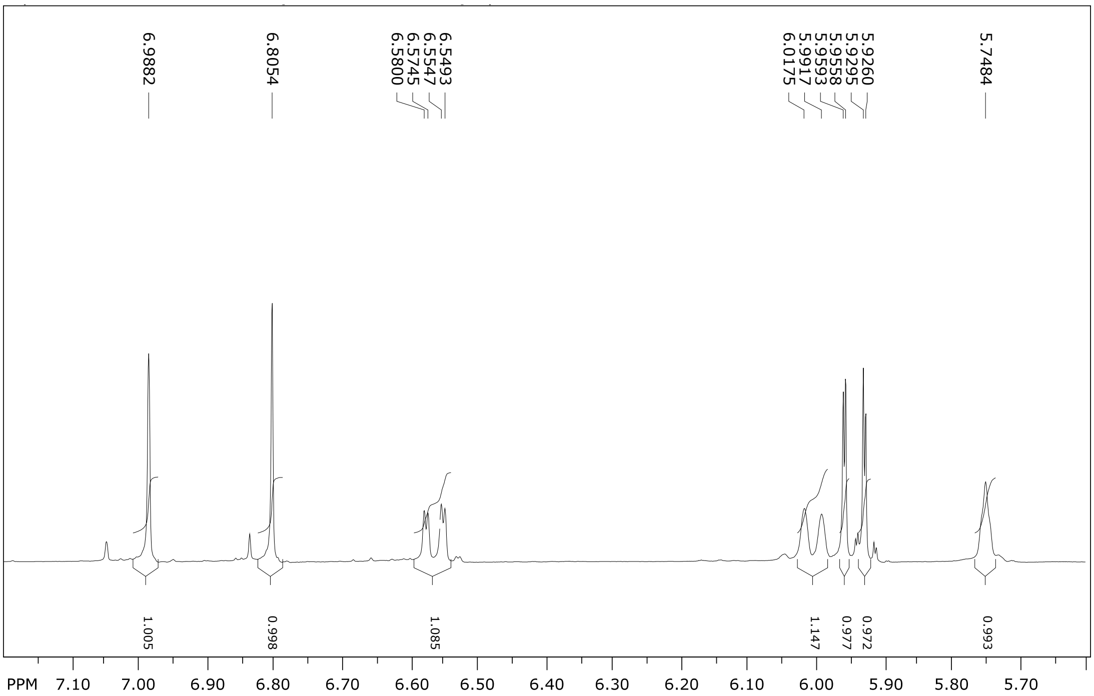

Figure S13. ^{13}C-NMR spectrum of erythrinine (**7**) [$CDCl_3$, 100 MHz].

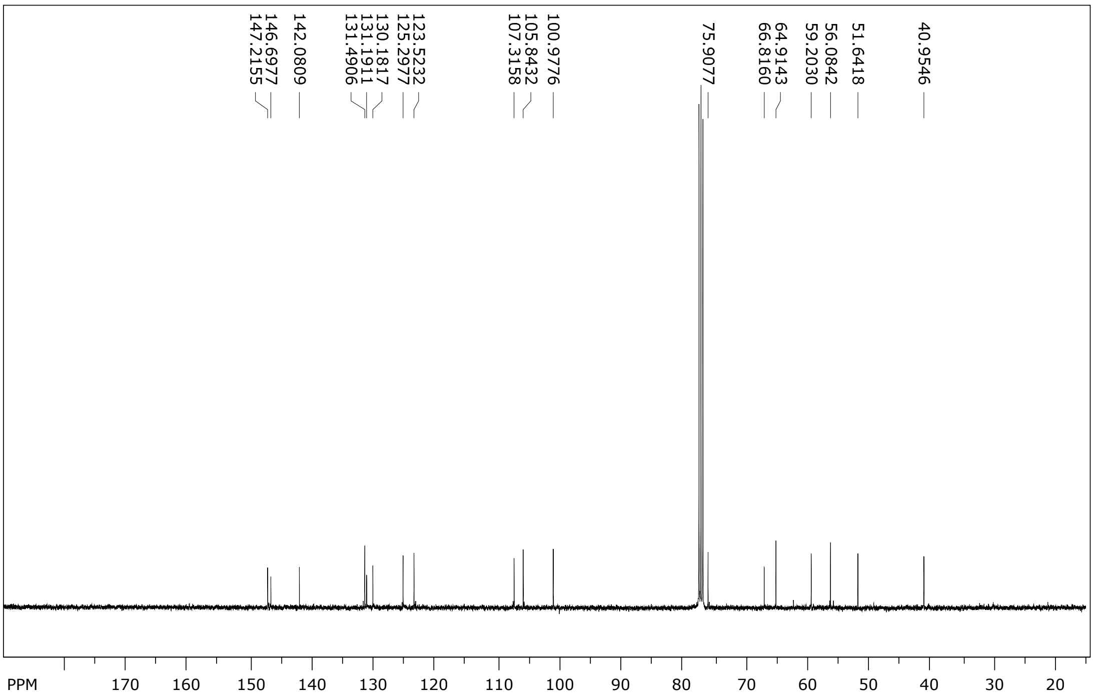

Figure S14. DEPT 135° spectrum of erythrinine (**7**) [$CDCl_3$, 100 MHz].

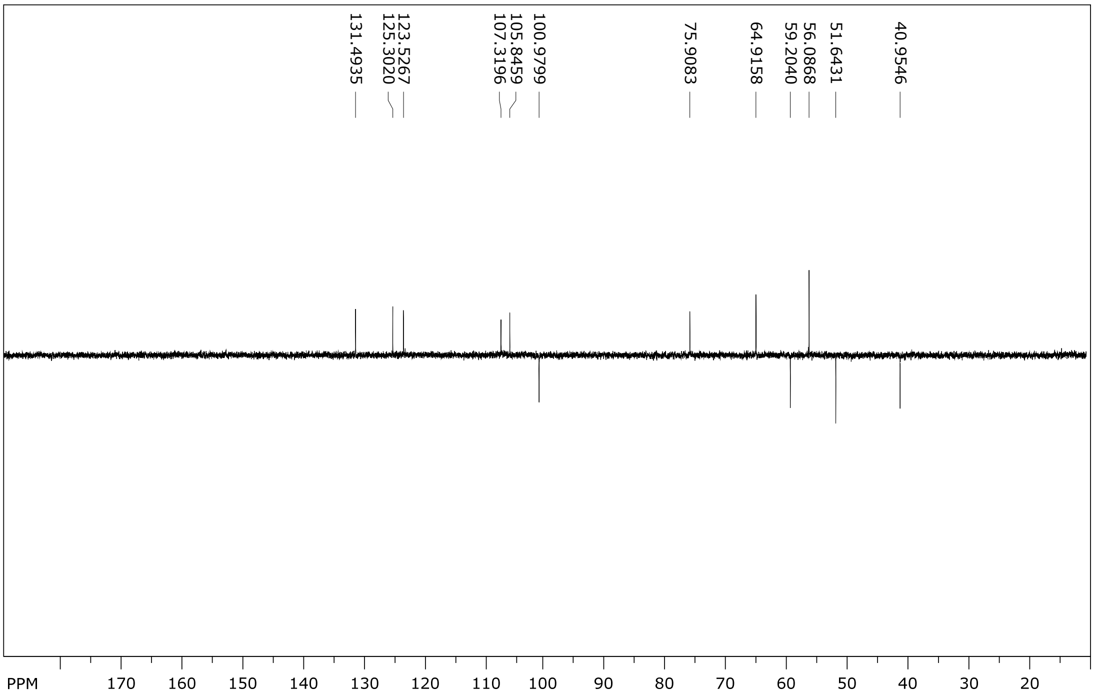

Figure S15. HRESI spectrum (positive ionization) of erythrinine (**7**).

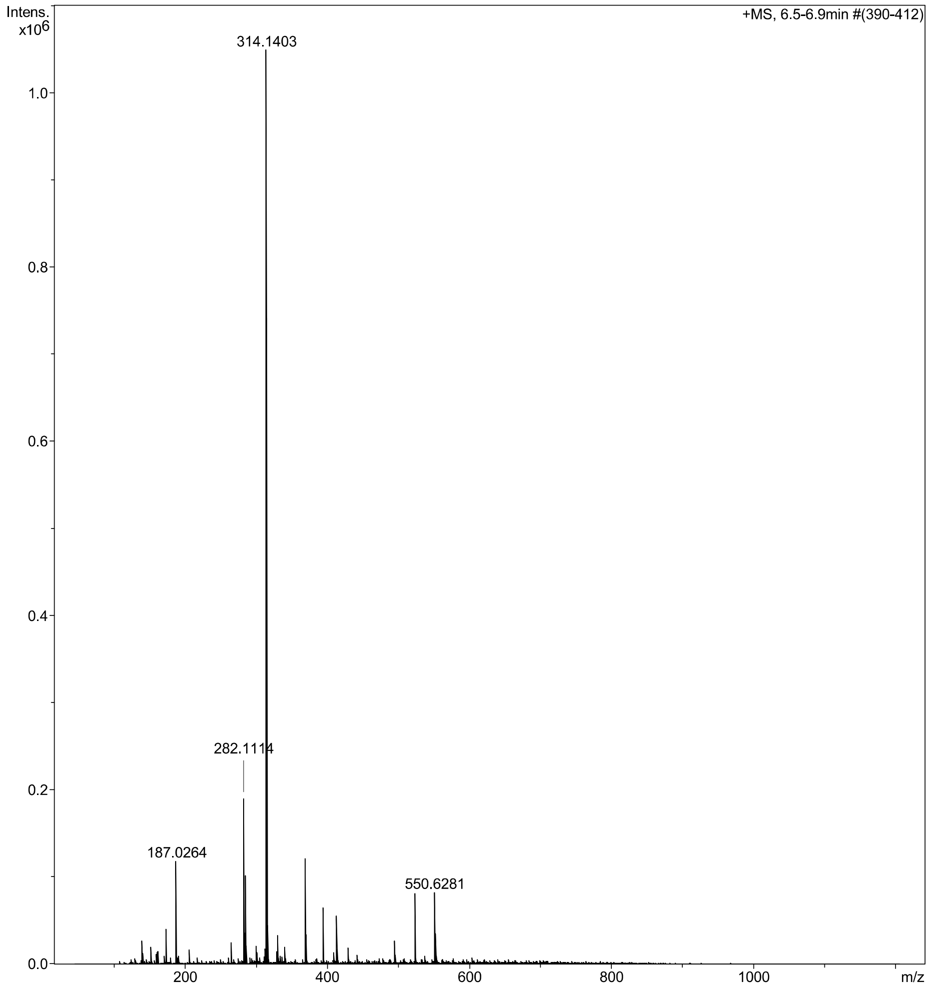

Figure S16. ^{1}H-NMR spectrum of erysovine (**8**) [$CDCl_3$, 400 MHz].

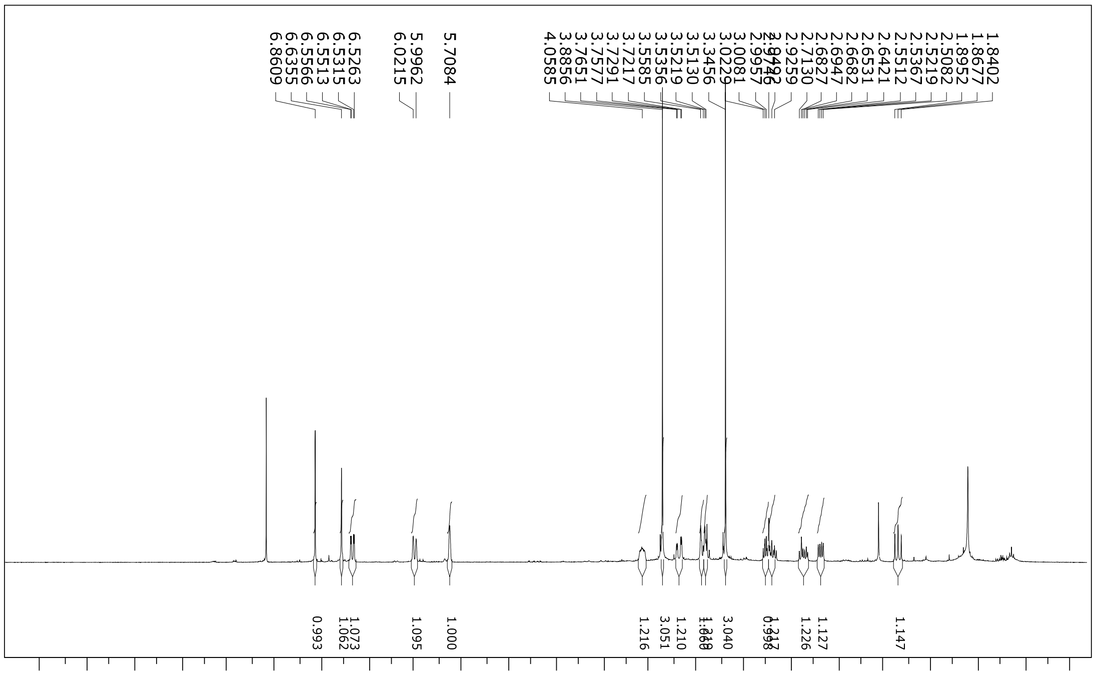

Figure S17. ^{1}H-NMR spectrum of erysovine (**8**) [$CDCl_3$, 400 MHz].

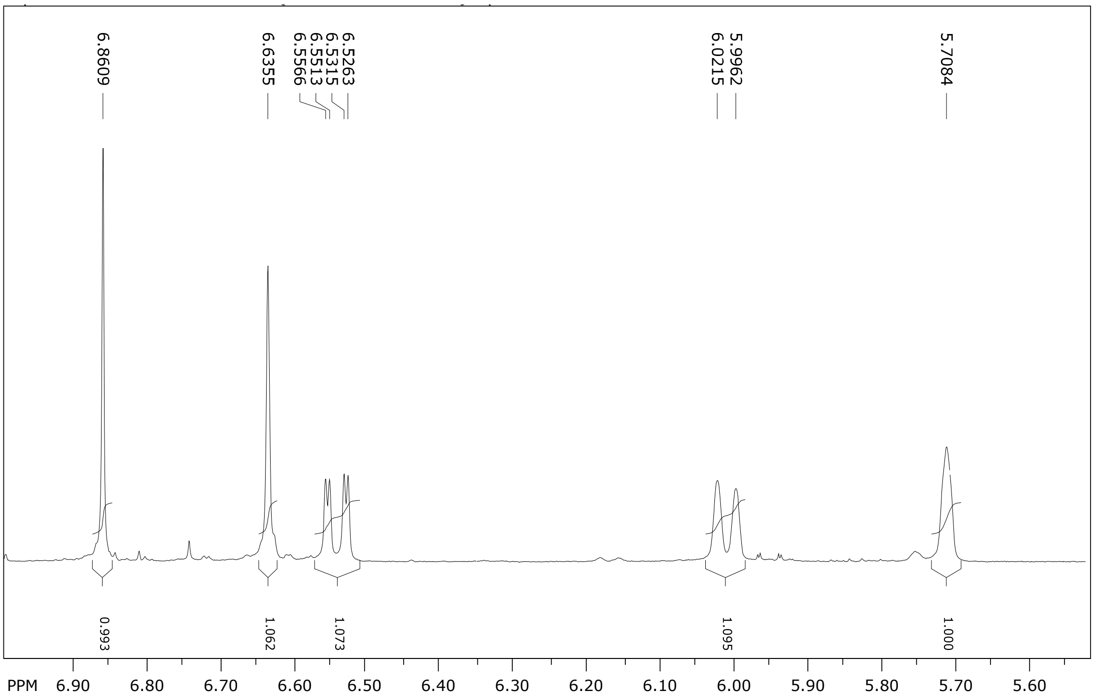

Figure S18. ^{13}C-NMR spectrum of erysovine (**8**) [$CDCl_3$, 100 MHz].

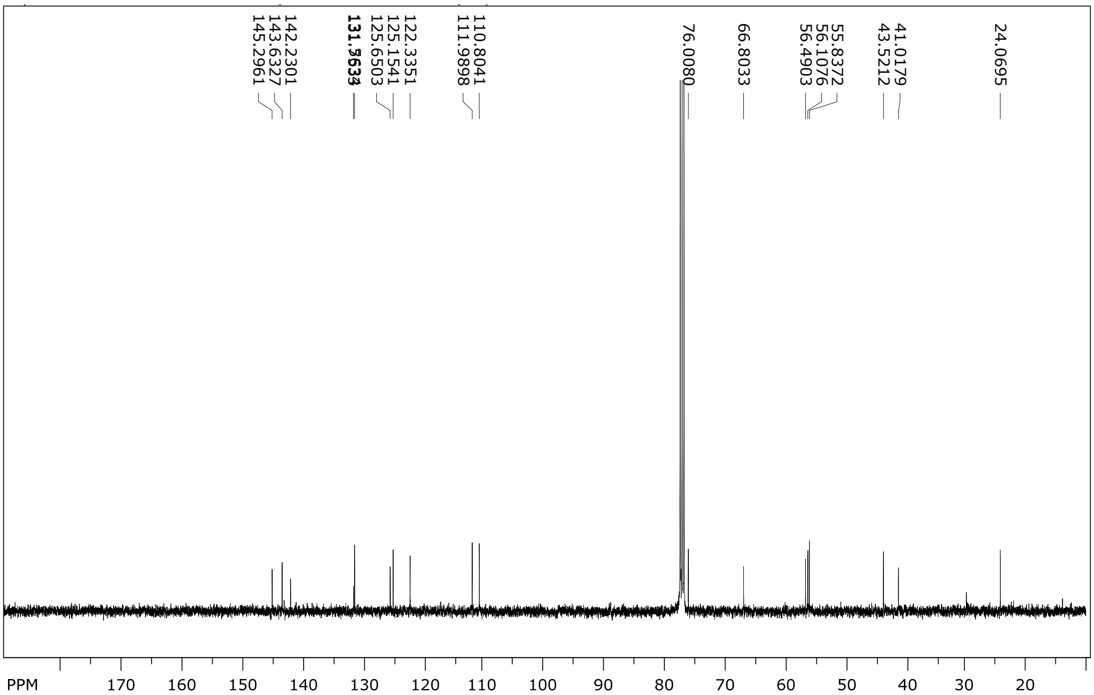

Figure S19. DEPT 135 ° spectrum of erysovine (**8**) [$CDCl_3$, 100 MHz].

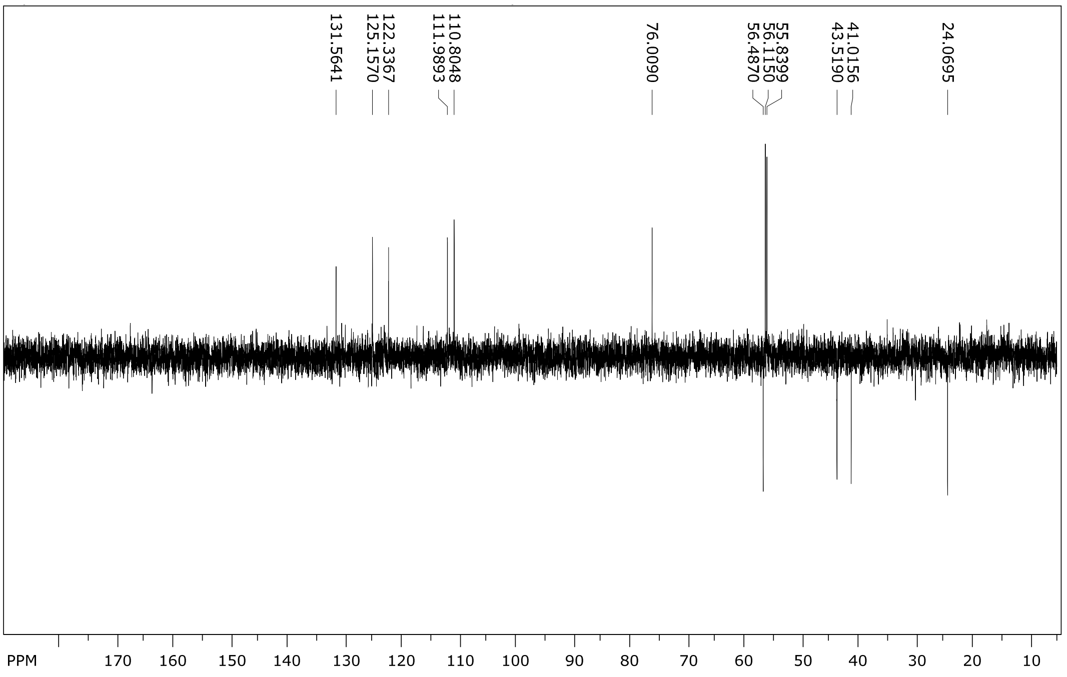

Figure S20. HMBC (8 Hz) contour map of erysovine (**8**).

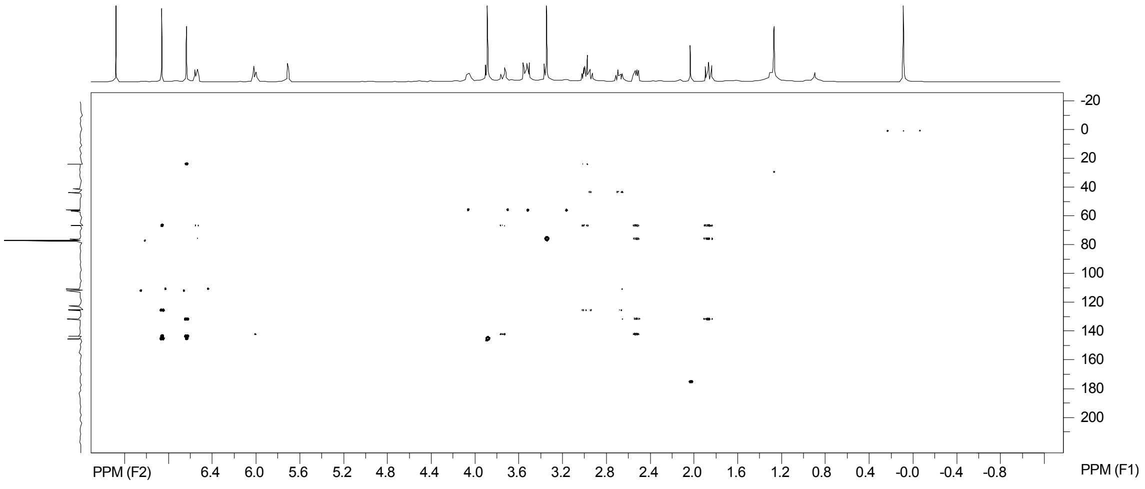

Figure S21. HMBC (8 Hz) contour map of erysovine (**8**).

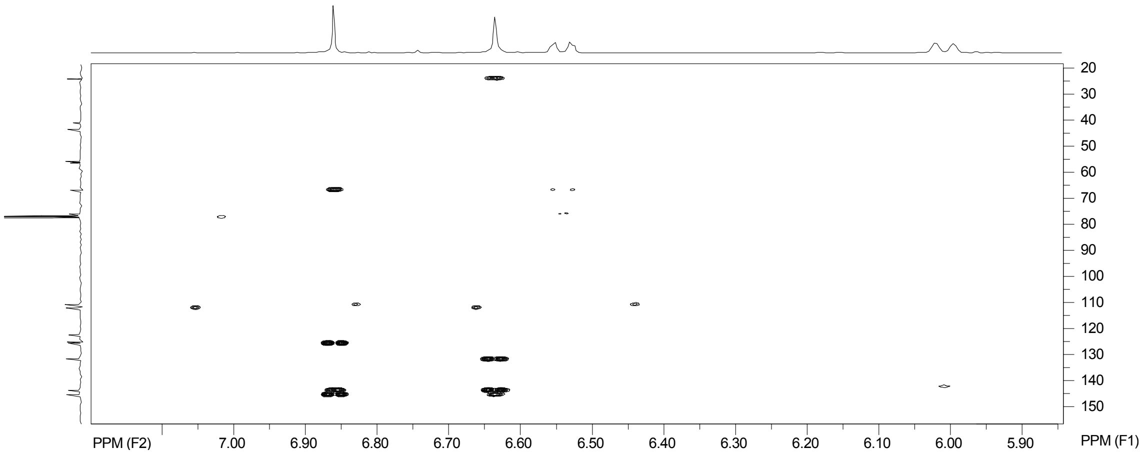

Figure S22. NOESY contour map of erysovine (**8**).

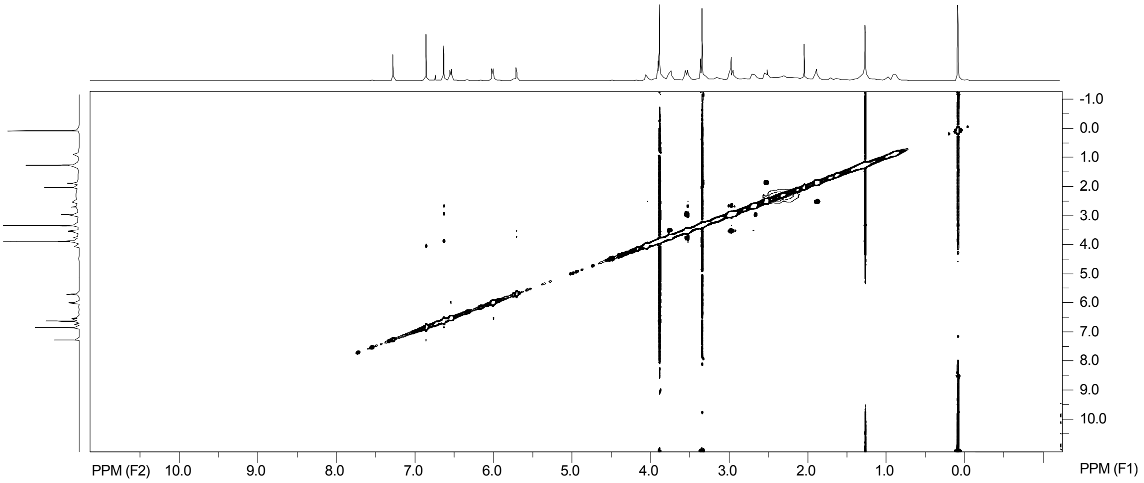

Figure S23. NOESY contour map of erysovine (**8**).

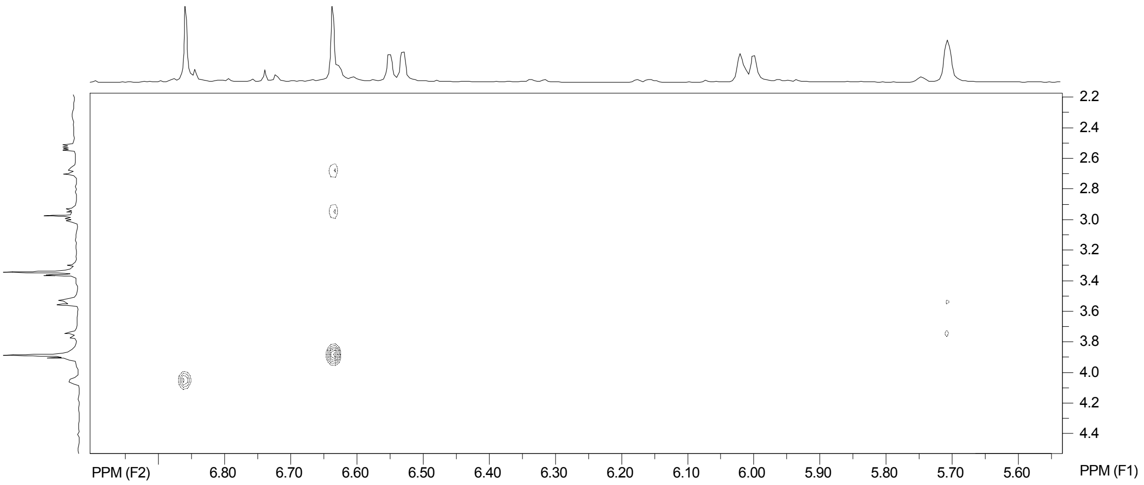

Figure S24. HRESI spectrum (positive ionization) of erysovine (**8**).

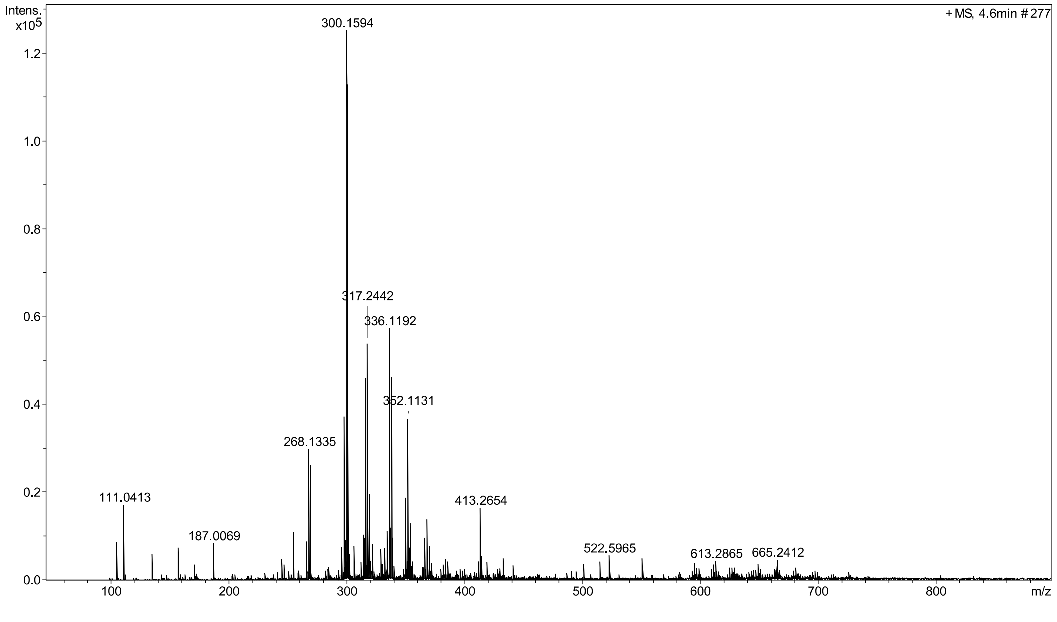

Figure S25. ^{1}H-NMR spectrum of erythratidinone (**9**) [$CDCl_3$, 400 MHz].

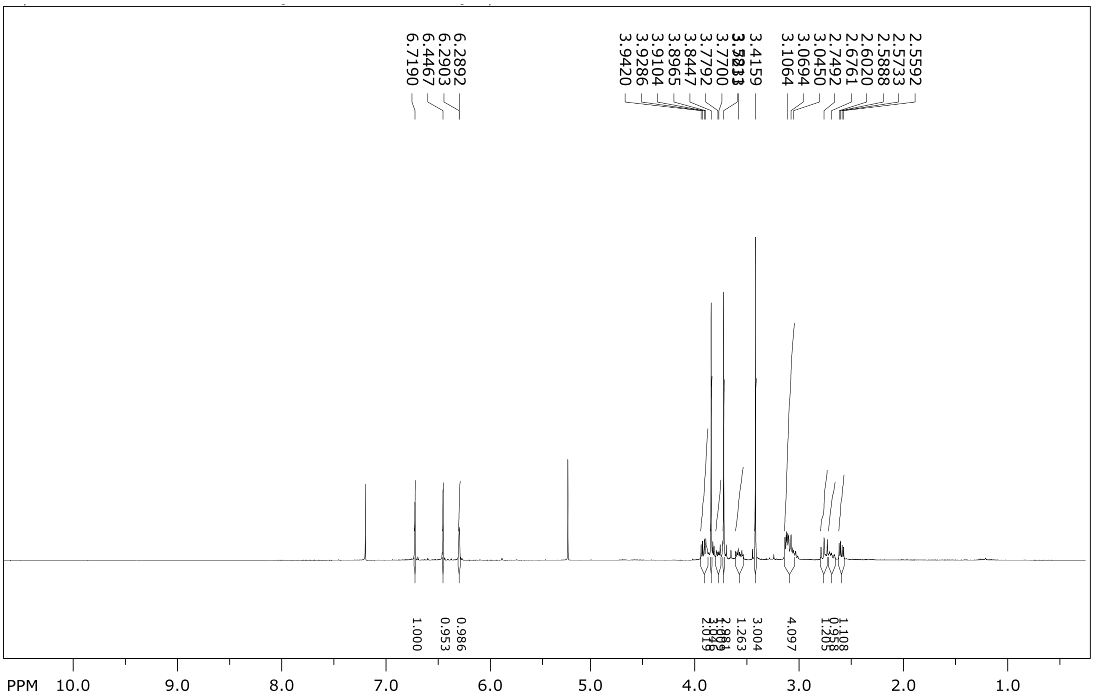

Figure S26. ^{1}H-NMR spectrum of erythratidinone (**9**) [$CDCl_3$, 400 MHz].

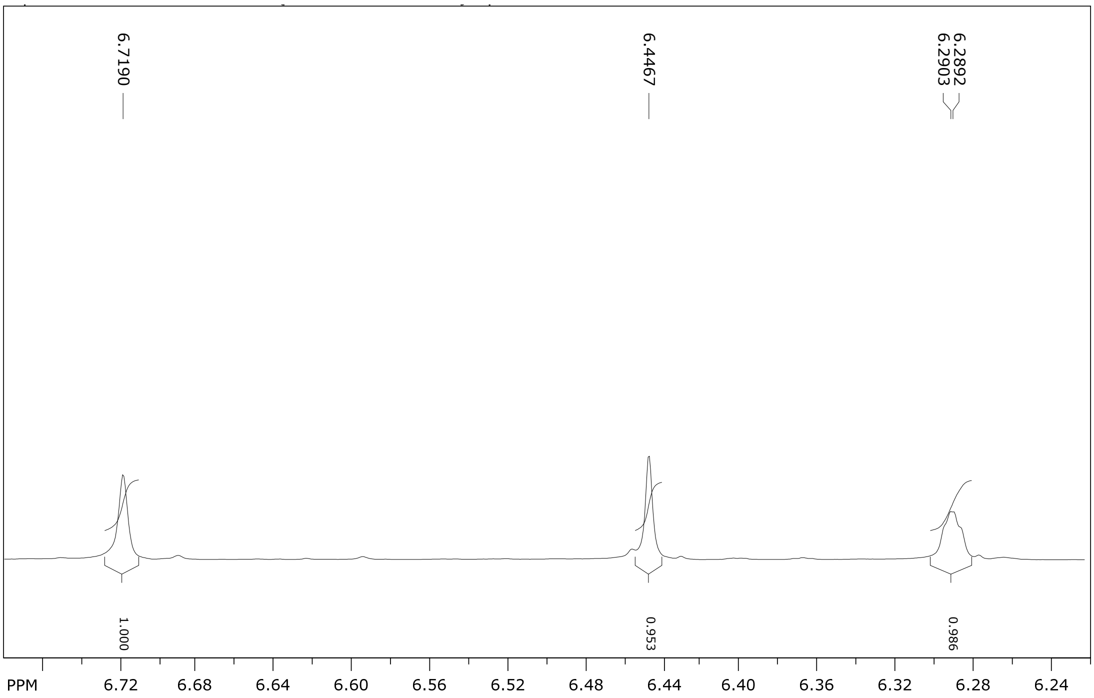

Figure S27. ^{13}C-NMR spectrum of erythratidinone (**9**) [$CDCl_3$, 100 MHz].

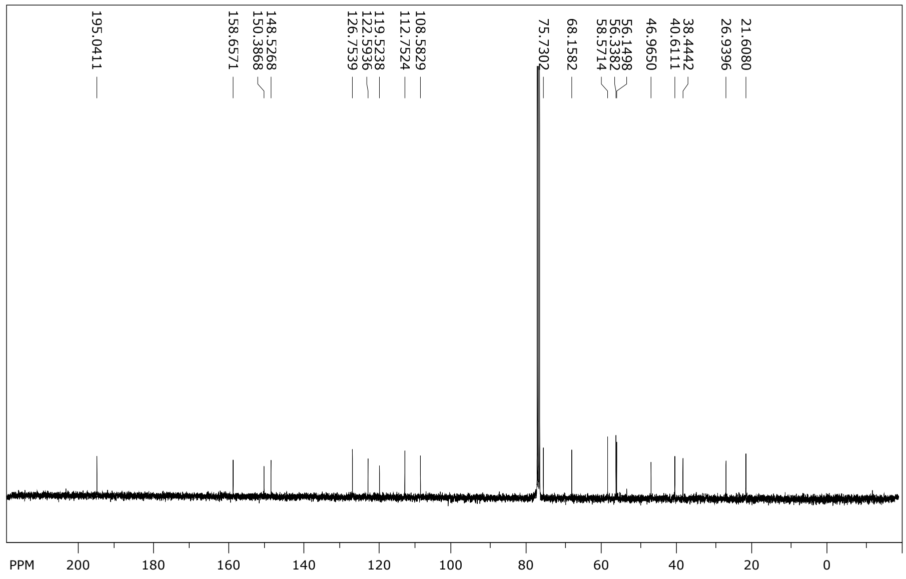

Figure S28. DEPT 135° spectrum of erythratidinone (**9**) [$CDCl_3$, 100 MHz].

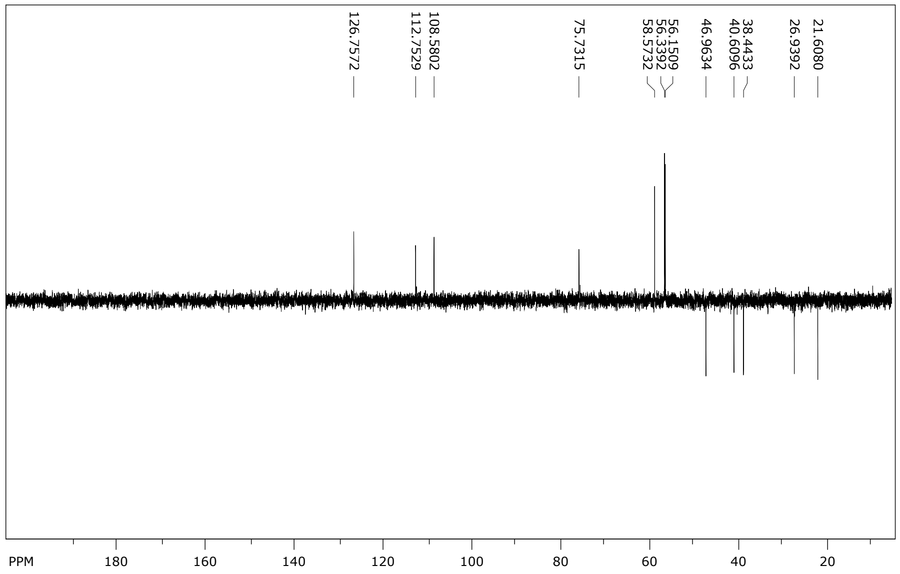

Figure S29. HMQC contour map of erythratidinone (**9**).

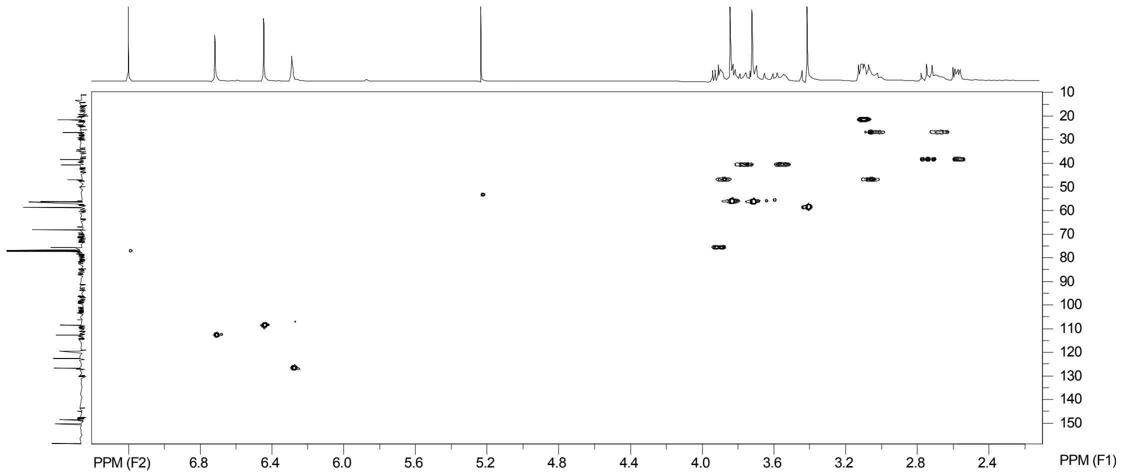

Figure S30. HMBC (8 Hz) contour map of erythratidinone (**9**).

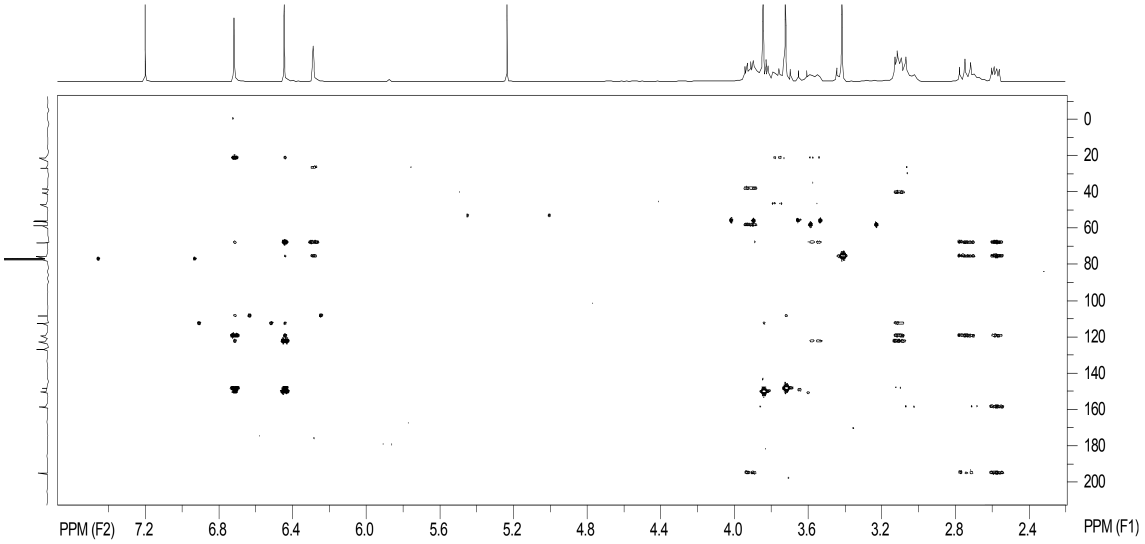

Figure S31. HMBC (8 Hz) contour map of erythratidinone (**9**).

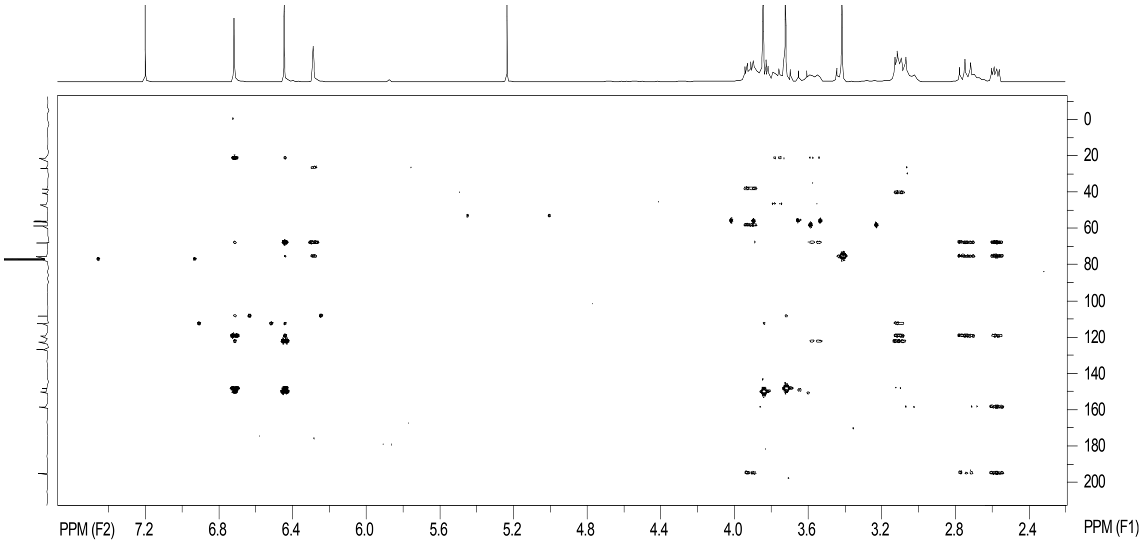

Figure S32. HRESI spectrum (positive ionization) of erythratidinone (**9**).

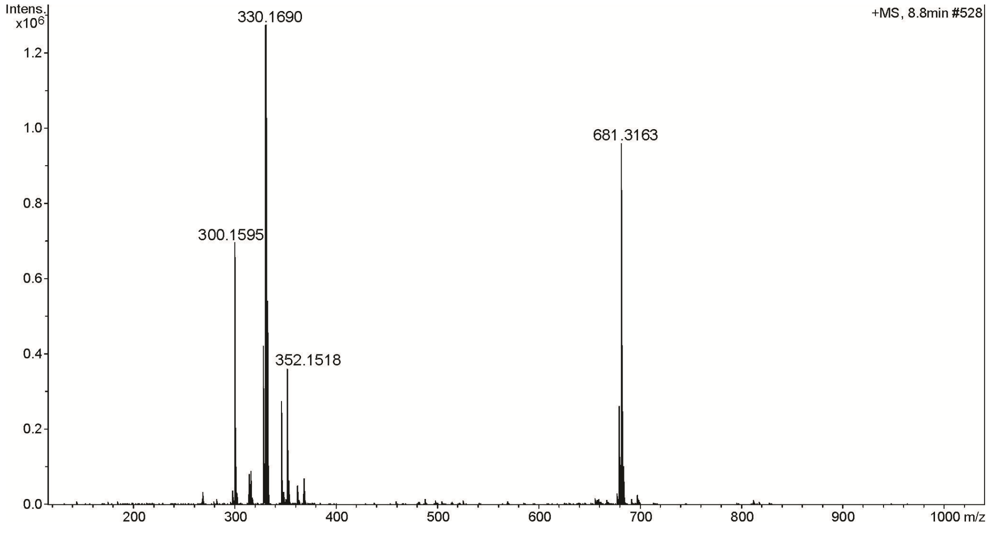

Supplement: Supplementary file 1 [file molecules-19-05692-s001.pdf]
